# Clinical Molecular Investigation of *Encephalitozoon cuniculi* in Pet Rabbits: Early Detection of Potential Risk for Animal and Human Infection

**DOI:** 10.1002/vms3.71081

**Published:** 2026-08-19

**Authors:** Maruša Škrbec, Brigita Slavec, Nina Kočar, Katarina Modrijan, Urška Kuhar, Marko Kolenc, Alenka Dovč, Olga Zorman Rojs, Jožko Račnik

**Affiliations:** ^1^ Institute of Poultry Birds, Small Mammals, and Reptiles Faculty of Veterinary Medicine University of Ljubljana Ljubljana Slovenia; ^2^ Institute of Microbiology and Parasitology Department of Bacteriology and Mycology Faculty of Veterinary Medicine University of Ljubljana Ljubljana Slovenia; ^3^ Institute of Microbiology and Immunology Laboratory for Syndromic Diagnostics and Molecular Bacteriology Faculty of Medicine University of Ljubljana Ljubljana Slovenia

**Keywords:** clinical signs, *Encephalitozoon cuniculi*, genotype, molecular detection, pet rabbit, urine

## Abstract

**Background:**

*Encephalitozoon cuniculi* is an obligate intracellular parasite belonging to the phylum Microsporidia, family Encephalitozoonidae. The parasite is distributed worldwide and infects a variety of mammals, with rabbits being the main host. Currently, the most reliable diagnostic method is serological testing, but various molecular techniques are also being used.

**Objective:**

This study assesses the presence, genotypes and clinical signs of *E. cuniculi* infection in pet rabbits in Slovenia, using molecular and serological methods.

**Methods:**

From January 2022 to 2024, urine samples from random pet rabbits (*n* = 247) were collected for polymerase chain reaction (PCR) testing and transmission electron microscopy (TEM) to search for the presence of *E. cuniculi* genetic material or spores.

**Results:**

With the real‐time PCR, *E. cuniculi* genetic material was detected in 37 of 247 (14.98%) rabbit urine samples. Serological testing revealed specific immunoglobulin M (IgM) and/or immunoglobulin G (IgG) antibodies in 91 of 133 rabbits (68.42%). A quarter of the serologically positive rabbits (25.27%) and almost half of the molecularly positive rabbits (43.24%) were clinically healthy and showed no signs of encephalitozoonosis. *E. cuniculi* genetic material in urine was mostly discovered in rabbits with signs of urinary tract disorders (27.03%) or neurological clinical signs (21.62%). Genotype I of *E. cuniculi* was detected in all rabbits. Despite the detection of the pathogens’ DNA, no *E. cuniculi* spores were observed by TEM.

**Conclusion:**

*E. cuniculi* infection is common among pet rabbits in Slovenia, with a high seroprevalence and detectable urinary shedding of parasite DNA. The presence of clinically healthy but infected rabbits indicates that subclinical infections are frequent. Genotype I appears to be the predominant genotype in the studied population. Combined molecular and serological testing may improve the detection and epidemiological characterization of *E. cuniculi* infections in rabbits.

## Graphical abstract

This study assesses the presence, genotypes and clinical signs of *E. cuniculi* infection in pet rabbits in Slovenia. Molecular analysis reveals the presence of *E. cuniculi* DNA (Genotype I) in 14.98% rabbit urine samples, mostly in rabbits with signs of urinary tract disorders. Quarter of the serologically positive rabbits and almost half of the molecularly positive rabbits were clinically healthy and showed no signs of encephalitozoonosis, presenting a possible infection risk for other rabbits, animals and people.

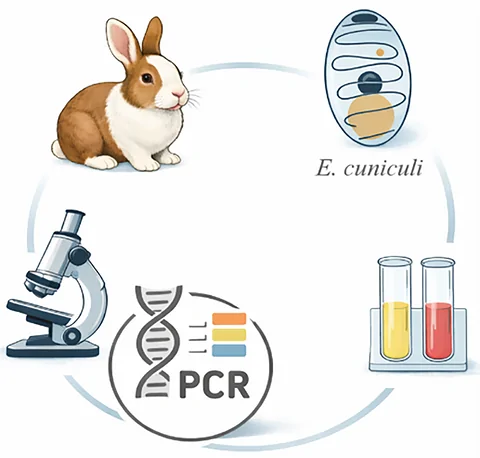

## 1 Introduction

*Encephalitozoon cuniculi* is an obligate intracellular spore‐forming protozoan parasite in the class Microsporidia (Morsy et al. 2020). The pathogen can infect various domestic and wild mammal species, and even some birds and reptiles can serve as reservoirs for these microsporidia (Magalhães et al. 2022; Kvapil et al. 2021). Even though *E. cuniculi* has a wide host distribution, it primarily affects domestic rabbits (*Oryctolagus cuniculus*), which are the main host. *E. cuniculi* is also considered an opportunistic pathogen in immunocompromised people (AIDS and cancer patients, transplant recipients, children, elderly people, travellers, etc.), workers in veterinary medicine, rabbit breeders and, importantly, pet rabbit owners (Magalhães et al. 2022; Dobosi et al. 2022). The main clinical signs detected in humans with microsporidia are fever of unknown origin, gastrointestinal problems, and various ocular, nasal and respiratory symptoms in addition to non‐specific signs (anorexia and weight loss) (Abu‐Akkada et al. 2015; Sak et al. 2011).

Clinical signs of encephalitozoonosis in rabbits vary. The most recognized clinical manifestation is vestibular disease (head tilt, nystagmus, circling, rolling or imbalance) with certain other central nervous system signs such as tremors, weakness of the hind legs and changes in the behaviour of the animal (aggression, deafness, fear or clumsiness; Künzel et al. 2008; Künzel and Joachim 2010). Rabbits may also display signs of renal failure (polyuria, polydipsia or anorexia) or other urinary system signs; for example, urinary sludge, incontinence, dysuria or urine scalding (Rich 2010; Harcourt‐Brown 2004). Especially young rabbits with encephalitozoonosis can suffer from phacoclastic uveitis (showing as cataract, uveitis or granuloma in the eyes; Özkan et al. 2018). There are also reports of various non‐specific disease presentations such as recurrent gastrointestinal hypomotilities, chronic weight loss, hepatitis and myocarditis (Škrbec et al. 2023; Özkan et al. 2018; Orcutt et al. 2020). In some rabbits, the infection is latent and may never manifest itself clinically (Varga Smith 2023b).

Pathogen's spores are excreted mostly with the urine of the infected rabbit into the environment. Infection with *E. cuniculi* mostly occurs with ingestion or inhalation of the spores. If the rabbit is latently infected and asymptomatic, his infection can go undetected, whereas the excretion and spreading of the spores into the environment (cages, food, water bowls, etc.) is present. These rabbits can be a source of infection for other rabbits, animals and humans (Latney et al. 2014).

Clinical diagnosis of encephalitozoonosis can be difficult because a fair number of rabbits do not display any clinical signs despite being infected. Therefore, more dependable assays for confirmation of specific antibodies against *E. cuniculi* are used to determine whether the animal is infected. A positive immunoglobulin G (IgG) titre reflects previous exposure to the pathogen, but it offers no indication of whether the disease is active. Furthermore, positive immunoglobulin M (IgM) and IgG titres suggest that infection is active but does not necessarily prove that clinical signs are present due to *E. cuniculi* (Jeklova et al. 2010). In human medicine, the use of polymerase chain reaction (PCR) is the standard methodology for detection of microsporidia in urine, faecal and tissue samples. In veterinary medicine, mostly urine or faeces samples are used for molecular detection of the pathogen in rabbits (Keeble 2016).

On the basis of the differences in the nucleotide sequences of the internal transcribed spacer (ITS) region of the ribosomal RNA (rRNA) gene, *E. cuniculi* strains can be divided into four genotypes. The sequence 5′‐GTTT‐3′ repeats three times in Genotype I strains, twice in Genotype II strains, four times in Genotype III strains and, finally, five times in the ITS region in Genotype IV strains (Magalhães et al. 2022). Genotype I (rabbit strain) is mainly detected in isolates from rabbits, Genotype II (murine strain) in rodents, Genotype III (canine strain) in dogs and Genotype IV (human strain) in people (Brdíčková et al. 2020). In addition, in humans, all four *E. cuniculi* genotypes have been confirmed, proving a zoonotic potential (Magalhães et al. 2022). Although genotyping allows the identification of strains with a certain host preference, recent studies have demonstrated that *E. cuniculi* has a low host specificity (Javadzade et al. 2021).

To date, there is no information about *E. cuniculi* genotypes in rabbits in Slovenia. There is also no overview of how many undiagnosed pet rabbits are examined at veterinary clinics and therefore present a potential risk of infection for other animals, veterinary staff and owners. The aim of this study was to determine the approximate incidence of asymptomatic rabbits that are infected with *E. cuniculi* and live in close contact with their owners or other companion animals. Furthermore, the reliability of serological indirect immunofluorescence assay (IFA) and molecular real‐time PCR testing was assessed and compared. Finally, clinical signs of rabbits with simultaneously specific antibodies against *E. cuniculi* and the pathogen's genetic material in their urine samples were determined and statistically processed to confirm whether there is a correlation between different types of clinical signs, especially urinary system clinical signs, and spore excretion.

## 2 Materials and Methods

### 2.1 Animals

All 247 pet rabbits, included in the study from January 2022 to 2024, were companion animals living in the same household as their owners. The median age of the animals was 4 years (IQR = 2–6), with the youngest rabbit being 6 months old and the oldest being 13 years old. The median weight of the study population was 2 kg (IQR = 1.5–2.4). All rabbits were mixed breed.

All rabbits underwent a prompt clinical examination. All 247 examined rabbits had a sample of their urine taken for PCR testing. If a rabbit showed any suspicious clinical signs of encephalitozoonosis, or if the owners wanted preventive serological testing, sera were also collected for an *E. cuniculi* antibody serology test (indirect IFA, *n* = 133).

Rabbits with PCR‐confirmed *E. cuniculi* genetic material in their urine (*n* = 37) were divided into groups on the basis of clinical signs. Group 1 (*n* = 16) was asymptomatic. The rabbits in this group were clinically healthy and did not show any clinical signs of *E. cuniculi* infection; they had mostly been brought to the clinic for a yearly check‐up or for spaying. The animals in Group 2 (*n* = 8) showed typical neurological clinical signs of *E. cuniculi*; for example, head tilt, imbalance, circling, weakness of the hind legs, aggression and fear. Rabbits with urinary system signs of *E. cuniculi* were assigned to Group 3 (*n* = 10); they showed one or more of the following clinical signs: dysuria, urine scalding, incontinence, urolithiasis, nephroliths or urinary sludge, or signs of chronic kidney disease. Group 4 (*n* = 0) was considered for rabbits with ocular encephalitozoonosis, showing as phacoclastic uveitis with or without cataract. Finally, Group 5 (*n* = 3) involved rabbits with atypical *E. cuniculi* clinical presentation, such as chronic weight loss, cachexia and recurrent gastrointestinal hypomotilities without a known reason.

### 2.2 DNA Extraction and PCR Amplification

Urine samples (1–2 mL) from 247 rabbits were collected into a sterile container with gentle manual pressure on the urinary bladder. The samples were stored at −70°C until the molecular testing was conducted. The protocol described by Javadzade et al. (2021) was used for preparing the samples for DNA extraction. Briefly, 300 µL of thawed urine was transferred into 1.5 mL tubes and centrifuged at 10,000 × *g* for 5 min, and the supernatant was discarded. The pellet was washed with 1 mL phosphate‐buffered saline (PBS) (pH: 7.5) with vigorous vortexing and centrifuged again under the same conditions. Total DNA was extracted from the pellet using the QIAamp DNA Mini Kit (Qiagen, Hilden, Germany) according to the manufacturer's instructions for DNA purification from tissues with a final elution volume of 100 µL (Çetinkaya et al. 2018).

For detecting *E. cuniculi*, we used the primers mps3 (5′ ggaattcacaccgcccgtcactat 3′), msp4a (5′ ccaagcttatgcttaagtccaaggggt 3′) and msp4b (5′ ccaagcttatgcttaagtccagggag 3′) to amplify the 3′ end of the small subunit rRNA, the ITS and the 5′ end of the large subunit rRNA (Zietek et al. 2014). Real‐time PCR with SYBR Green 1 dye and ROX passive reference dye was performed using the Quantstudio 5 instrument (Thermo Fisher Scientific Inc., Europe).

We prepared 15 µL of PCR reaction using 7.5 µL of 2X Maxima SYBR Green/ROX qPCR Master Mix (2X) (Thermo Scientific, Dreieich, Germany), 0.4 µM of each PCR primer, 2 µL of isolated DNA and deionized water up to 15 µL. Cycling parameters included an initial denaturation step at 95°C for 10 min, followed by 50 cycles at 95°C for 60 s, 58°C for 60 s and 72°C for 90 s, followed by one cycle of melt curve stage at 95°C for 15 s, 72°C for 60 s and 95°C for 1 s. For positive control, the DNA isolate extracted from *E. cuniculi* Whole Cell Antigen Suspension (Medicago, Uppsala, Sweden) was used.

### 2.3 Sequences

All PCR products with a melting temperature (*T*_m_) between 79°C and 83°C and detectable Ct values were run on a 1.8% agarose gel (Sigma‐Aldrich, USA) and electrophoresed at 130 V for 30 min. The gel was stained with MIDORI Green Advance (NIPPON Genetics EUROPE, Düren, Germany), and the bands were visualized using the BioRad Gel Doc 2000 Imaging System and Quantity One 4.4.0 Analysis Software. Bands of approximately 300 bp in length were excised from the gel and purified using the FastGene Gel/PCR Extraction Kit (Nippon Genetics, Europe, Düren, Germany). The PCR products were sent to the Eurofins Genomics laboratory (Eurofins Genomics, Ebersberg, Germany) for Sanger sequencing.

Nucleotide sequence assembly and analysis was performed using Geneious Prime Software Suite v.2022.1.1 (Biomatters Ltd., Auckland, New Zealand). The assembled sequences were compared with the nucleotide sequences of *E. cuniculi* deposited in GenBank using BLASTn. Nucleotide sequences of relevant *E. cuniculi* strains were retrieved from GenBank. The nucleotide alignments of the *E. cuniculi* strains from this study and the strains from GenBank were generated using the MAFFT program (Kato and Standley 2013). Because all nucleotide sequences of *E. cuniculi* from this study were identical, only one was deposited in GenBank, under accession number PV854194.

### 2.4 Detection of Antibodies

Rabbits that showed potential signs of encephalitozoonosis were serologically tested for the presence of specific anti‐*E. cuniculi* IgM and/or IgG antibodies. An intravenous catheter was used for the collection of approximately 0.5 mL of blood from the marginal ear vein. BD Microtainer tubes containing Clot Activator/SST Gel were used for the blood collection (Becton, Dickinson and Company, Franklin Lakes, NJ, USA). The blood sample was centrifuged at 2000 rpm for 10 min. Next, sera were collected and tested for the presence of specific antibodies. Serological diagnosis was carried out with the indirect IFA, using a commercial test kit (MegaFLUO, *E. cuniculi*, Megacor, Austria). Anti‐rabbit IgM and IgG antibodies, conjugated with fluorescein isothiocyanate (FITC), were added (Megacor, Austria). Each serum was tested and titrated from 1:40 to 1:1280. Conjugates were added according to the manufacturers’ instructions.

### 2.5 Transmission Electronic Microscopy (TEM)

Urine samples that tested positive for *E. cuniculi* by molecular methods and exhibited the lowest cycle threshold (Ct) values were further examined by TEM to detect the presence of *E. cuniculi* spores. The samples were centrifuged at 500 × *g* to concentrate potential spores. The resulting sediment was resuspended in PBS, and formvar‐coated copper grids were incubated with the suspension to allow sample adsorption. Negative staining was performed using 1% uranyl acetate. The grids were examined using a TEM (JEM‐1400 Plus; JEOL, Tokyo, Japan) operated at 120 kV. Samples were assessed for the presence of potential *E. cuniculi* spores; these were identified on the basis of their morphology, with a typical size ranging between 0.8 and 1.0 µm.

### 2.6 Statistical Analysis

Normality of the distribution was assessed using the Shapiro–Wilk test, complemented by histograms and *Q*–*Q* plots. Given the mixed distributional characteristics, summary statistics are presented as medians and interquartile ranges. Disease prevalence was calculated for each clinical group and the total sample. The fourth group, with a sample size of only two, was excluded from inferential statistical analyses. Between‐group differences in PCR positivity rates were assessed using Fisher's exact test. In addition, Cramer's *V* was calculated to assess the effect size of these differences. The Kruskal–Wallis test was used for comparing CT values across groups, with epsilon squared used to determine the effect size. To assess diagnostic agreement between PCR and IFA in the subset of rabbits tested with both methods (*n* = 133), a confusion matrix was constructed. Sensitivity, specificity and predictive values for PCR were calculated using IFA as the reference comparator. Cohen's kappa statistic was used to quantify inter‐method agreement, and McNemar's test was applied to evaluate the asymmetry of discordant test results. The data were analysed using R version 4.3.3 and Jamovi project (2024, version 2.5). Significance was set at *p* < 0.05.

## 3 Results

With PCR, 37/247 (14.98%) rabbit urines were tested positive for the presence of *E. cuniculi* DNA. Blood samples for serological testing were taken from 133/247 rabbits; of these, 91 (68.42%) animals had confirmed IgG and/or IgM antibodies against *E. cuniculi*. Seventeen animals (15.31%) simultaneously tested molecularly and serologically positive for *E. cuniculi* infection. The distribution of rabbits according to their serological and molecular testing results is summarized in Table S1.

Some rabbits were unfortunately not tested with the IFA method to confirm possible presence of IgG and/or IgM (*n* = 114/247; 46.15%), as their owners did not give consent for testing or the rabbits did not have a clinical indication for blood drawing. Serological status of these 114 rabbits was therefore not known. However, with use of PCR, 20 rabbit urine samples in this group contained *E. cuniculi* genetic material, meaning infection was discovered by chance.

Forty‐two rabbits (42/133) were tested seronegative; therefore, they were excluded from further statistical analysis because our main object of study was *E. cuniculi* infected rabbits. Their addition to the first part of the statistical presentation was made to allow insight into the approximate disease incidence in the population of Slovenian rabbits and to confirm the credibility of PCR test results for seronegative rabbits.

**FIGURE 1:**
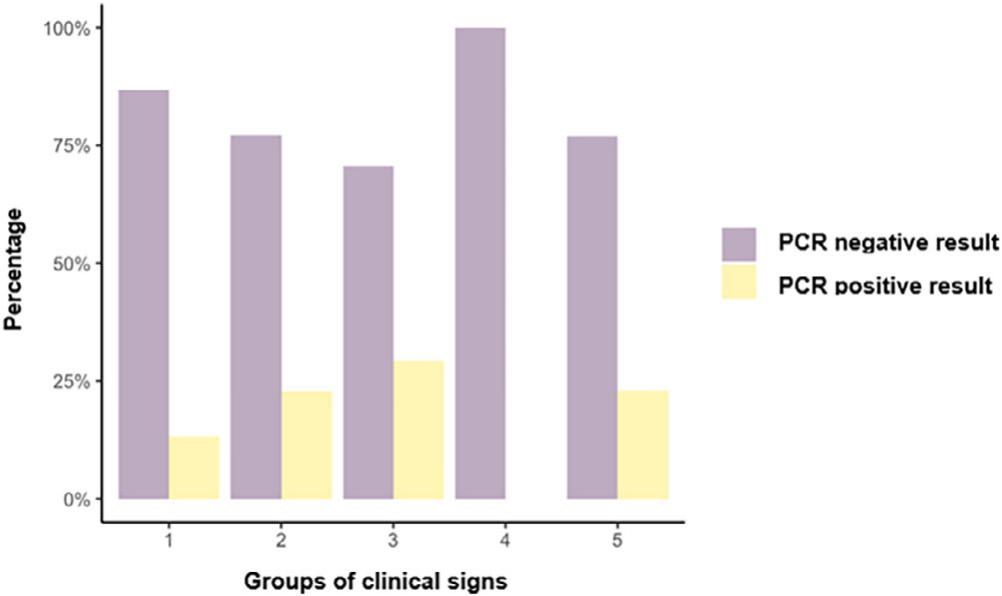
Graphic presentation of different groups of clinical signs (1–5) and their percentage of positive or negative PCR results. Group 1 is clinically healthy, asymptomatic rabbits. Group 2 is rabbits with neurological clinical signs (i.e., head tilt, nystagmus, imbalance, circling, weakness of the hind legs and change in behaviour). Group 3 consists of rabbits with urinary system signs (i.e., dysuria, urine scalding, incontinence, urolithiasis, nephroliths, urinary sludge and signs of chronic kidney disease). Group 4 (ocular encephalitozoonosis) has no representatives. Finally, Group 5 is represented by rabbits with atypical *Encephalitozoon cuniculi* clinical signs (i.e., chronic weight loss, cachexia and recurrent gastrointestinal hypomotilities without a known reason). PCR, polymerase chain reaction.

The 37 rabbits for which molecular testing confirmed the presence of *E. cuniculi* DNA in their urine were divided into five groups, depending on their clinical signs. The distribution of PCR‐positive and PCR‐negative rabbits across the different clinical groups is shown in Figure 1. Group 1 (*n* = 16; 43.24%) was dedicated to rabbits that did not show any clinical signs of encephalitozoonosis. These animals were mainly discovered to be infected by coincidence; most of them were not serologically tested for *E. cuniculi*, and only PCR confirmed the presence of the pathogen DNA in their urine. Their owners brought them into the clinic for preventive testing and a general health check, various stomatology procedures, neutering or some other clinical signs unrelated to encephalitozoonosis (respiratory distress and infections, dermatological problems, etc.). In this group 2 rabbits were also simultaneously tested serologically. One rabbit had elevated both IgG and IgM antibodies, whereas the other had only elevated IgG. Group 2 (*n* = 8; 21.62%) consisted of animals with sudden onset of central nervous signs attributed to *E. cuniculi*, such as imbalance, rolling in circles or repetitive falling to one side, head tilt, nystagmus, weakened hind legs and aggressive behaviour. Seven rabbits concurrently tested seropositive. Six of them (75%) had elevated both IgG and IgM, and one animal (12.5%) had only IgG. Group 3 (*n* = 10; 27.03%) was assigned to rabbits showing clinical signs associated with urinary tract disorders (urolithiasis, nephrolithiasis, urinary sludge and distended urinary bladder, incontinence, dysuria or urine scalding). Again, most (*n* = 7) had also serologically confirmed *E. cuniculi* infection, and the other three were discovered to be infected by chance, with molecular testing. All seropositive rabbits had both IgG and IgM antibodies present. Group 4 (ocular encephalitozoonosis) had no representatives. Finally, Group 5 consisted of three rabbits (8.11%) that were either chronically losing weight, despite normal appetite, or were suffering from repetitive idiopathic gastrointestinal hypomotilities. One case was seropositive for *E. cuniculi*, and the other two were identified by chance. The seropositive rabbit had elevated both IgG and IgM antibodies. An overview of the sex, serological status, clinical status (clinical sign group) and Ct values of the 37 rabbits in which *E. cuniculi* DNA was detected in urine samples by real‐time PCR is provided in Table S2.

With TEM we did not find any structures that resembled *E. cuniculi* spores in morphology or size in the urine samples of 37 rabbits for which molecular testing showed the presence of *E. cuniculi* DNA.

**FIGURE 2:**
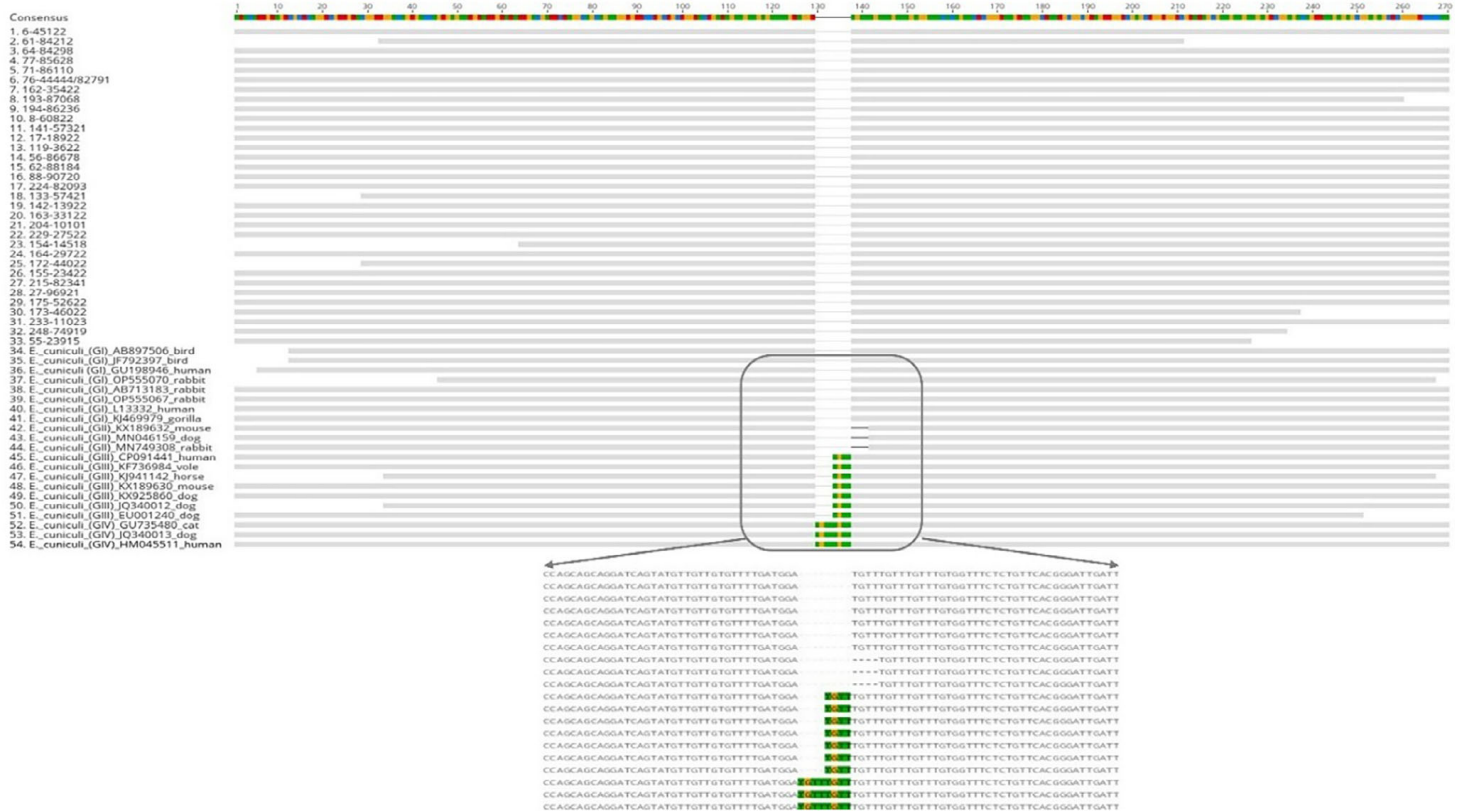
The nucleotide alignment of our rabbit population's strains (Genotype I) and selected *Encephalitozoon cuniculi* strains from all four genotypes, which have different 5′‐GTTT‐3′ repeats.

On the basis of the BLASTn results, the *E. cuniculi* strains from this study belong to Genotype I. This was confirmed by nucleotide alignment of our strains and selected *E. cuniculi* strains from all four genotypes, which have different 5′‐GTTT‐3′ repeats (Figure 2). The strains from this study were all identical and had three sequence 5′‐GTTT‐3′ repeats.

The serological status of the 91 seropositive rabbits showed that most of them (*n* = 61; 67.03%) had concurrently elevated IgM and IgG antibodies against *E. cuniculi*. A quarter (*n* = 23; 25.27%) only had specific IgG antibodies elevated, whereas a small number of rabbits (*n* = 7; 7.69%) tested positive for only IgM antibodies. Among the 17 animals that were simultaneously molecularly positive and seropositive, the ordering/organization of serological status was similar. The majority of these rabbits (*n* = 15; 88.24%) had both IgG‐ and IgM‐specific antibodies present, and two rabbits (11.76%) showed only elevated IgG‐specific antibodies in their sera. There was no elevation of only IgM antibodies in this group. Furthermore, an overview of the serological status of rabbits from different groups of clinical signs also shows somewhat comparable results.

Different groups of clinical signs among the molecularly positive rabbit population were compared to determine whether there was a difference in the ratio of positive to negative PCR test results. Fischer's exact test revealed no significant differences (*p* = 0.84), with a Cramer's *V* of 0.07 (95% CI = [0.03–0.32]), indicating a very weak association between the groups of patients and the PCR test results. Given the non‐significant *p* value, the effect size, as measured by Cramer's *V*, likely reflects only a negligible association.

In addition, statistical analysis was carried out to determine whether any of the clinical sign groups had lower CT values in comparison to others. The Kruskal–Wallis test was used to assess differences between the patient groups with regard to CT values, yielding a test statistic of *χ* ^2^(2) = 2.678, *p* = 0.262. The effect size estimated by epsilon squared (*ε* ^2^ = 0.03, 95% CI = [0.01–0.14]) indicated a small effect. Together with the non‐significant result of the Kruskal–Wallis test, this suggests that any differences between the groups are likely negligible and not statistically significant.

The melting point of the PCR products obtained ranged from 81.1°C to 82.0°C, with a median value of 81.65°C.

Finally, to compare the performance of PCR and IFA in detecting *E. cuniculi* in rabbits, we conducted a comparative analysis on the subset of animals tested with both methods. Although neither of the methods represents a definitive gold standard, IFA was used as a reference method to evaluate the classification characteristics of PCR. Out of the 133 rabbits tested with both methods, PCR identified 17 IFA‐positive rabbits as positive and showed no false positives among IFA‐negative cases, resulting in a high specificity (100%) and positive predictive value (PPV = 100). However, PCR failed to detect infection in the majority of IFA‐positive rabbits, yielding a low sensitivity (18.7%) and overall detection rate (12.8%). The comparison of PCR and IFA test results is presented in Table S3. To assess agreement between the methods, Cohen's kappa was calculated and indicated only slight agreement (*κ* = 0.13, 95% CI [0.062–0.19]), reflecting low overlap between PCR and IFA detection. Furthermore, the significant McNemar's test (*p* < 0.001) indicates a substantial asymmetry in test outcomes. As seen in the matrix, there is a pronounced excess of IFA‐positive/PCR‐negative cases compared to PCR‐positive/IFA‐negative cases. In summary, whereas PCR is highly reliable for ruling out infection, it performs poorly in detecting positive cases.

## 4 Discussion

This study examines the commonness of *E. cuniculi* among Slovenian pet rabbits and, more importantly, the approximate incidence of asymptomatic infected rabbits examined at the clinic. This study showed that almost half (44.94%) of the rabbit study population was infected with *E. cuniculi*. With PCR, 14.98% rabbit urines were tested positive for the presence of *E. cuniculi* DNA, and with IFA 36.84% rabbits had confirmed IgG and/or IgM antibodies against *E. cuniculi*. Almost half (Group 1, 43.24%) of rabbits that had *E. cuniculi* genetic material detected in their urine by PCR were asymptomatic or at least not showing any clinical signs related to encephalitozoonosis at the clinic. These animals (e.g., their urine) present a potential risk of infection for other animal patients and veterinary staff at the clinic, and potentially for owners at home. If a rabbit is suffering from a latent *E. cuniculi* infection, it may be asymptomatic and appear clinically healthy while excreting infective *E. cuniculi* spores into the environment. Spores of *E. cuniculi* are mainly excreted with the urine of the infected animal for approximately 35 days post‐infection and for up to 3 months (Jeklova et al., 2020; Dobosi et al. 2022). After 3 months, shedding of spores becomes irregular/intermittent throughout the infected rabbit's life (Künzel and Fisher 2018). Excreted spores are greatly resistant to environmental changes and can survive outside the host up to 6 weeks at 22°C in dry conditions, and for several months under humid conditions (Latney et al. 2014; Santaniello et al. 2021). Therefore, ingestion or inhalation of spores present in the environment is possible for other rabbits, animals and humans (Latney et al. 2014). Especially veterinary workers and owners, who are in daily close physical contact with potentially infected rabbits and their excretions, cages and equipment, can be at a higher risk of zoonotic infection, because *E. cuniculi* spores are highly environmentally persistent and resistant to many disinfectants. An effective measure to reduce the potential exposure of animals and humans to *E. cuniculi* spores is regular cleaning and disinfection of animal housing facilities with bleach and 70% ethanol (Magalhães et al. 2022).

Most of the studied rabbit population (especially Group 1) that had *E. cuniculi* genetic material detected in their urine samples were mainly discovered as infected by chance, meaning they were asymptomatic on the day of the clinical examination. Other molecularly positive rabbits were presented to the clinic with either typical signs of an *E. cuniculi* infection, such as neurological clinical signs (Group 2, 21.62%) and urinary system disorders (Group 3, 27.03%), or some atypical clinical presentation of chronic wasting, cachexia or repetitive episodes of gastrointestinal hypomotilities of unknown origin (Group 5, 8.11%). To our knowledge, no study obtained detailed groups of clinical signs of encephalitozoonosis, specifically in rabbits with PCR‐confirmed DNA material of *E. cuniculi* in urine. Furthermore, there is also no information on whether rabbits in one group of clinical signs are more likely to shed infective spores in their urine than other groups of clinical signs. The intention of our study was to analyse whether rabbits with a renal form of encephalitozoonosis (Group 3) are more likely to have *E. cuniculi* DNA material detected in their urine because the predilection organ of *E. cuniculi* in their cases was presumably the kidneys. In an infected animal, *E. cuniculi* has a predilection for the brain, kidneys and heart, although heart disease due to *E. cuniculi* is rarely diagnosed. In the target organs, chronic inflammation is caused by the presence of the pathogen and the host's immune system response. This results in the development of granulomatous lesions that affect normal tissue anatomy and physiology. In the kidneys, non‐purulent interstitial nephritis and fibrosis develop, possibly leading to chronic renal failure (Harcourt‐Brown 2002). The microsporidian spores are mostly located in renal vessels and glomerular capillaries, in the tubular epithelium or renal interstitium, and/or free in the collecting tubules (Özkan et al. 2018; Harcourt‐Brown and Holloway 2003). Presumably, if there are more *E. cuniculi* spores in the targeted kidney tissue, more damage to the organ occurs and more *E. cuniculi* DNA could be released and detected with PCR in the urine. Even though PCR‐positive rabbits with urinary system signs related to *E. cuniculi* (Group 3) constituted the largest group by clinical signs, the statistical analysis shows no significant differences between detected *E. cuniculi* DNA in urine samples from Group 3 and urine samples in other groups of clinical signs. One factor contributing to these results could be the small number of animals in different research groups. Further investigations with larger groups of animals and potentially different PCR kits and protocols would be needed to disprove this.

The vast majority of rabbits (88.24%) that were simultaneously *E. cuniculi* seropositive and had the pathogen's DNA detected in their urine with PCR showed elevations of both types (IgM and IgG) of specific immunoglobulins against *E. cuniculi*, meaning that the rabbits were suffering from an active ongoing infection, even if not showing any clinical signs. In these cases, appropriate therapy (e.g., anthelmintic fenbendazole or albendazole) should be administered to at least temporarily prevent spore excretion in the urine (Santaniello et al. 2021). On the other hand, just a few rabbits (11.76%) had only IgG antibodies elevated, which is usually seen in a latent infection, which could potentially become clinical should the balance between host and parasite change (Varga Smith 2023a).

On the basis of the statistical comparison and analysis, PCR detection of *E. cuniculi* DNA material in the urine was less effective in providing additional data for diagnosis of encephalitozoonosis than confirmation of the infection with specific anti‐*E. cuniculi* antibodies in the rabbits’ sera. This finding was also confirmed in other studies. Baz‐González et al. (2022) stated that PCR is suitable for detecting microsporidia during active infection, whereas spores are being shed but could lead to underestimating the prevalence due to intermittent/irregular spore‐shedding periods, such as at the beginning of the primary infection or in chronic infections. When interpreting PCR results, it is also important to note that PCR spore detection relies on the dose‐dependent spore burden within the patient (Künzel et al. 2008; Mancinelli 2015). Consequently, the number of undetected *E. cuniculi* infections among our rabbits and the general rabbit population is possibly significantly higher. In the future, urine excretion rates should be studied in chronically infected rabbits to ascertain and potentially improve PCR urine spore detection. On the other hand, diagnostic aspect of PCR detection of *E. cuniculi* DNA in urine samples is worthwhile and can be considered beneficial in evaluating possible acute onsets of spore excretion in non‐clinical infected rabbits and can consequently clarify the decision if the patient needs treatment. Namely, in rabbits with elevated IgM (e.g., elevation of only IgM or both IgM and IgG) with or without clinical signs of encephalitozoonosis, treatment with an appropriate antiparasitic is indicated, as a possible active infection and spore shedding are present. Detected elevation of only IgG, which is commonly found in clinically asymptomatic infected rabbits, only confirms the presence of antibodies at the particular time of testing, indicating past exposure to the parasite; it does not reflect the course of clinical disease, because carrier status may occur. As serum IgG titres can remain elevated for years without any clinical signs, the treatment protocol for these patients sometimes remains unclear (Keeble 2016). In our study the majority of rabbits with detected *E. cuniculi* material in their urine had elevated both IgM and IgG antibodies. In these patients treatment is indicated not only because of IgM elevation and active parasite replication but also because of possible excretion of infectious *E. cuniculi* material/spores into the environment. However, a small number of infected rabbits had elevated only IgG antibodies alongside with *E. cuniculi* DNA in their urine. According to past literature data (only) IgG‐positive rabbits do not need treatment, especially if being asymptomatic (Keeble 2016). Nevertheless, as treatment with benzimidazoles (fenbendazole and albendazole) limits spore formation and therefore their excessive excretion (Varga Smith 2023a), therapy would be comprehended also in only IgG‐positive rabbits with detected *E. cuniculi* DNA in their urine. In that context, conformation of *E. cuniculi* genetic material in the urine of rabbit patients with (only) IgG titre elevations can guide the clinician's decision to start with the treatment despite the absence of clinical signs.

Despite the presence of *E. cuniculi* genetic material in some urine samples, we did not find any structures that resembled *E. cuniculi* spores in morphology and/or size with TEM. The key factor contributing to these findings could be the low sensitivity of TEM, especially in combination with the given high CT values. Furthermore, freezing the samples at −70°C could affect the morphology and quantity of the mature spores, making them difficult to recognize. For further studies, direct immunofluorescence (DIF) microscopy with a higher sensitivity could be considered.

In rabbits, the most frequent genotype of *E. cuniculi* is Genotype I, also known as the ‘rabbit strain’, even though some authors have also reported the presence of Genotypes II and III (Deng et al. 2020; Valenčakova et al. 2012). In our study, all *E. cuniculi* isolates were identified as Genotype I. This genotype was also reported by various studies in some European countries, the United States, Australia and elsewhere (Javadzade et al. 2021; Kimura et al. 2013). In addition to rabbits, Genotype I has also been reported in dogs (Wang et al. 2018), birds (Pirestani et al. 2013) and humans (Tavalla et al. 2017). Therefore, zoonotic concern due to transmission of *E. cuniculi* Genotype I, particularly from pet rabbits, seems to once again be an important issue.

## 5 Conclusion

In conclusion, the findings of this survey show that the *E. cuniculi* infection rate among Slovenian pet rabbits is quite high (44.94%). Although many rabbits show typical signs of encephalitozoonosis, almost half of them appear asymptomatic. Almost one‐fifth of the examined rabbit patients were clinically healthy, even though they probably shed *E. cuniculi* spores into the environment as there was evidence of *E. cuniculi* DNA in their urine. These *E. cuniculi* undiagnosed animals present the greatest risk of infection to other rabbits and animals at the veterinary clinics, as well as to veterinary staff and owners. In addition to asymptomatic rabbits (43.24%), the pathogen's DNA is excreted to the greatest extent by rabbits with signs of urinary tract disorders (27.03%) or neurological clinical signs (21.62%). Most of the rabbits with urinary tract disorders (70%) and neurological signs (75%) have elevated both IgM and IgG antibodies, meaning they are suffering from an active ongoing infection. Therefore, biosafety education for veterinary healthcare workers and owners should be encouraged, and personal hygiene should be ensured for those that handle the animals and their excretions. Although PCR detection of *E. cuniculi* genetic material in rabbit urine may present itself as less reliable diagnostic tool for clinicians to diagnose infected animals due to intermittent spore shedding, the method can still be beneficial for deciding whether to start treatment in an infected asymptomatic patient. The presence of *E. cuniculi* DNA in the urine sample of an asymptomatic rabbit with only elevated IgG titres could be a sign of an active infection and, therefore, an initiation for treatment. Additionally, regular PCR testing of urine could also be of great help in evaluating the reliability of fenbendazole effectiveness during or after therapy because the drug should cause reduction of spore shedding.

## Author Contributions

**Maruša Škrbec**: writing – original draft, conceptualization, methodology, investigation, formal analysis, resources. **Brigita Slavec**: writing – review and editing, methodology, investigation, validation, resources. **Nina Kočar**: writing – review and editing, investigation. **Katarina Modrijan**: writing – review and editing, investigation. **Urška Kuhar**: writing – review and editing, methodology, investigation. **Marko Kolenc**: writing – review and editing, methodology, investigation. **Alenka Dovč**: writing – review and editing. **Olga Zorman Rojs**: writing – review and editing, supervision, funding acquisition, project administration. **Jožko Račnik**: writing – review and editing, conceptualization, methodology, investigation, resources, funding acquisition, project administration. This manuscript has been reviewed by all authors.

## Funding

This study was supported by the Slovenian Research and Innovation Agency (research core funding no. P4‐0092 and junior researchers grant 55729).

## Ethics Statement

The authors confirm that the ethical policies of the journal, as noted on the journal's author guidelines page, have been adhered to. The Committee for Animal Welfare for Experimental Purposes of the University of Ljubljana's Faculty of Veterinary Medicine (certificate number: 7c1f86f4000000005751d8fa) assessed that the primary purpose of the study is verification of animals’ health status and treatment of animals by implementing good veterinary practice, and it deemed that permission for procedures on animals from the national ethics committee was not required. The Institute of Poultry, Birds, Small Mammals and Reptiles (Veterinary Faculty, Ljubljana, Slovenia) under which the University Clinic of Birds, Small Animals and Reptiles operates, allows the implementation of necessary diagnostic and therapeutic procedures on animals and the use of analysed data. The owners of the animals agreed to the use of the medical records and results for research purposes.

## Conflicts of Interest

The authors declare no conflicts of interest.

## Supporting information




**Supporting File 1**: vms371081‐supp‐0001‐SuppMat.docx

## Data Availability

The datasets presented in this article are not readily available due to client data privacy protection. Requests to access the datasets should be directed to the corresponding author.
