# Supplementary material for: Clinical Molecular Investigation of Encephalitozoon cuniculi in Pet Rabbits: Early Detection of Potential Risk for Animal and Human Infection
Source: Vet Med Sci. 2026 Aug 19;12(5):e71081. doi: 10.1002/vms3.71081 (PMC13489076; doi:10.1002/vms3.71081)
Supplement: Supplementary file 1 [file VMS3-12-e71081-s001.docx]

**Supplementary Material**

Table 1. *E. cuniculi* infection status for rabbits by serological and molecular testing.

| Method | *E. cuniculi* infection | | |
| --- | --- | --- | --- |
| Serological (IFA) | Positive:  91/247 | Negative:  42/247 | Not tested:  114/247 |
| Molecular (PCR, urine) | Of which positive: 17/91 | Of which positive: 0/42 | Of which positive: 20/114 |

Table 2. Overview of the sex, serological status, clinical status (i.e., clinical sign group) and CT value of the 37 rabbits that tested molecularly positive.

^a^ CT value – cycle threshold; the number of cycles needed to replicate enough DNA to be detected.

^b^ Group 1 – clinically healthy, asymptomatic rabbits.

^c^ Group 2 – rabbits with neurological clinical signs (i.e., head tilt, nystagmus, imbalance, circling, weakness of the hind legs, change in behavior, etc.).

^d^ Group 3 - rabbits with urinary system signs (i.e., dysuria, urine scalding, incontinence, urolithiasis, nephroliths, urinary sludge, signs of chronic kidney disease, etc.).

^e^ Group 5 - rabbits with atypical *E. cuniculi* clinical signs (i.e., chronic weight loss, cachexia, recurrent gastrointestinal hypomotilities without a known reason).

| Sex | Clinical sign group | Presence of IgM and IgG (serological status) | PCR result (urine) | CT^a^ |
| --- | --- | --- | --- | --- |
| M | 1^b^ | No information | Positive | 33.8 |
| M | 1 | No information | Positive | 33.2 |
| F | 1 | No information | Positive | 29.8 |
| F | 1 | No information | Positive | 29.9 |
| F | 1 | No information | Positive | 28.5 |
| M | 1 | No information | Positive | 36.6 |
| F | 1 | No information | Positive | 34.7 |
| F | 1 | No information | Positive | 29.8 |
| M | 1 | IgM and IgG | Positive | 35.5 |
| F | 1 | No information | Positive | 29.4 |
| M | 1 | No information | Positive | 36.4 |
| M | 1 | No information | Positive | 30.9 |
| M | 1 | No information | Positive | 34.9 |
| F | 1 | No information | Positive | 34.7 |
| M | 1 | No information | Positive | 31.9 |
| M | 1 | IgG | Positive | 31.9 |
| F | 2^c^ | IgM and IgG | Positive | 31.7 |
| M | 2 | IgG | Positive | 31.5 |
| F | 2 | IgM and IgG | Positive | 34.4 |
| M | 2 | IgM and IgG | Positive | 35.4 |
| M | 2 | IgM and IgG | Positive | 32.0 |
| F | 2 | IgM and IgG | Positive | 30.5 |
| F | 2 | IgM and IgG | Positive | 32.5 |
| M | 2 | No information | Positive | 33.7 |
| F | 3^d^ | IgM and IgG | Positive | 33.7 |
| F | 3 | IgM and IgG | Positive | 31.5 |
| M | 3 | IgM and IgG | Positive | 30.7 |
| F | 3 | IgM and IgG | Positive | 26.0 |
| M | 3 | IgM and IgG | Positive | 30.8 |
| M | 3 | No information | Positive | 31.4 |
| F | 3 | IgM and IgG | Positive | 28.7 |
| F | 3 | No information | Positive | 31.5 |
| M | 3 | IgM and IgG | Positive | 33.6 |
| F | 3 | No information | Positive | 33.3 |
| F | 5^e^ | IgM and IgG | Positive | 30.4 |
| M | 5 | No information | Positive | 31.7 |
| F | 5 | No information | Positive | 37.1 |

Table 3. A confusion matrix, showing the performance of each diagnostic method (IFA and PCR).

|  |  | IFA | |  |
| --- | --- | --- | --- | --- |
|  |  | Positive | Negative | Total: |
| PCR | Positive | 17 | 0 | 17 |
|  | Negative | 74 | 42 | 116 |
|  | Total: | 91 | 42 | 133 |
